# Quarantine-related depression and anxiety during coronavirus disease (COVID-19) outbreak

**DOI:** 10.1192/j.eurpsy.2021.1742

**Published:** 2021-08-13

**Authors:** D. Alateeq, S. Aljhani, M. Alsubaie, I. Althiyabi, S. Majzoub

**Affiliations:** 1 Clinical Sciences, Princess Nourah bint Abdulrahman University, Riyadh, Saudi Arabia; 2 Department Of Psychiatry, College Of Medicine, Qassim University, Riyadh, Saudi Arabia; 3 Collage Of Medicine, Princess Nourah bint Abdulrahman University, Riyadh, Saudi Arabia; 4 Department Of Psychiatry, Prince Mohammed Bin Abdulaziz Hospital, Riyadh, Saudi Arabia; 5 The National Transformation Program, Ministry of Health, Riyadh, Saudi Arabia

**Keywords:** Depression, quarantine, Anxiety, COVID-19

## Abstract

**Introduction:**

Psychological well-being has an important impact on individuals. In the face of the health threat of COVID-19, psychological changes as fear stress anxiety and depression is reported.

**Objectives:**

Explore the prevalence of depression and anxiety among people under quarantine during the COVID-19 outbreak in Saudi Arabia.

**Methods:**

A cross-sectional study of a convenience sample of 65 participants who were quarantined at multiple hotels under the supervision of the Saudi Ministry of Health. The patient health questionnaire (PHQ-9) and generalized anxiety disorder questionnaire (GAD-7) were used to assess depression and anxiety.

**Results:**

The majority of the participants were male (66.2%), aged 18–29 (47.7%) from the Eastern region (66.15%), who were still waiting for the result of the COVID-19 test (64.6%). Approximately half of the sample had depressive and anxiety symptoms (49.2% and 44.6%, respectively). The depression mean score was significantly higher only among the 18–29 age group. However, the depression and anxiety mean scores were higher among females than males and among participants with positive COVID-19 test results compared to those who had pending or negative results. The anxiety mean score was higher during the first week of quarantine, but the depression mean score was higher during later weeks.

**Conclusions:**

Depression and anxiety were prevalent among people in quarantine during the beginning of the COVID-19 outbreak in Saudi Arabia. It is crucial to study the most effective interventions to reduce the psychological consequences, especially for vulnerable groups. Longitudinal research studies need to be conducted to follow up regarding participants’ mental health symptoms and evidence-based interventions.

**Disclosure:**

No significant relationships.

